# Advanced signal-processing framework for remote photoplethysmography-based heart rate measurement: Integrating adaptive Kalman filtering with discrete wavelet transformation

**DOI:** 10.1371/journal.pone.0340097

**Published:** 2026-01-20

**Authors:** Uday Debnath, Sungho Kim

**Affiliations:** Department of Electronic Engineering, Yeungnam University, Gyeongsan, South Korea; Southeast University, CHINA

## Abstract

Remote photoplethysmography (rPPG) is a noncontact camera-based optical technique which analyses skin-color variations due blood volume changes beneath the skin and estimates vital signs such as heart rate (HR). Driven by the increasing demand for long-term unobtrusive vital sign monitoring systems, rPPG has grown significant interest in clinical and nonclinical domains. However, conventional rPPG methods are often limited by following challenges such as motion artifacts (MA), ambient light intensity and weak signal quality. Moreover, their overall accuracy is significantly impacted by demographic and skin-tone variations. To address these limitations, an advanced signal-processing framework integrating discrete wavelet transform (DWT) for denoising and signal-to-noise-ratio enhancement with residual-based adaptive Kalman filtering (RAKF) for frame-wise temporal consistency and MA reduction is proposed (DWT-RAKF). Further, a multi-channel fusion strategy is integrated with dual-stage band-pass filtering technique to isolate the HR signal while effectively discarding unrelated signal components. Our proposed framework is evaluated on both public and custom datasets. Regarding the PURE dataset, the proposed framework obtained a mean absolute error (MAE) and a root mean square error (RMSE) of 0.72 and 1.14 bpm respectively, outperforming several conventional state-of-the-art methods. To further evaluate its real-world performance, intra-dataset testing is implemented using custom dataset comprising subjects with varying skin-tones and under natural lighting conditions. The results revealed that the proposed algorithm obtained the lowest MAEs of 0.94 and 1.11 bpm for fair-skinned and dark-brown subjects respectively, indicating that the integration of the proposed signal filtering strategy with rPPG achieved effective real-time HR measurement.

## Introduction

Heart rate (HR) is among the most critical physiological parameters for determining overall health status during cardiovascular activity analysis. It has been widely used for the preliminary analysis of various cardiovascular and psychological disorders [1]. Conventional contact-based HR measurement approaches include the electrocardiogram (ECG) method which involves specific sensors attachment to the skin. Although these contact-based wearable sensors approaches can predict heart rate with high precision, they may cause discomfort or even induce allergic reactions in subjects, and this renders them unsuitable for long-term monitoring and nonclinical applications [3]. Smartwatches and other wearable devices use contact photoplethysmography (PPG) sensors which can also monitor heart rate continuously but they still require skin contact and may cause discomfort during prolonged wear. It also becomes challenging to achieve higher accuracy using PPG sensor due to signal deterioration from intense motion influence during physical movements [45]. Remote photoplethysmography (rPPG) provides a non-contact alternative particularly in clinical scenarios where attaching sensors to the skin become difficult to the patients, such as in neonatal care facilities [50], skin burn units [51] and infection-control units in the hospital [52]. However, the major drawback of rPPG method is that it is highly sensitive to ambient lighting variations and motion artifacts, and its performance has been validated under mild or moderate head movements only in previous studies [53]. Therefore, rather than aiming to replace contact-based sensors in dynamic scenarios, this study focuses on improving signal processing framework of rPPG method for physiological signal estimation in indoor environments where subject movement occurs naturally.

rPPG is an emerging optical technique for estimating HR using standard RGB cameras. Previous studies have demonstrated that rPPG can also be extended to estimate respiratory rate, blood oxygen saturation, and even blood pressure trends under different scenarios [46–48]. By leveraging ambient light, rPPG eliminates the direct contact requirement that is characteristic of conventional methods [1]. It captures subtle skin-color variations which correspond to blood volume fluctuations during heartbeats, thereby enabling contactless HR estimation from a distance. Generally, the camera captures both specular and diffuse reflections from skin area when recording the video. These specular reflections include surface data, whereas diffuse reflections provides spatial and temporal information which are crucial for accurately extracting physiological signals from video data [17]. To effectively measure HR, the rPPG system processes these reflections, detecting signal frequency peaks that correspond to individual heartbeats [18,19]. Conventional rPPG methods mainly rely on signal-processing techniques to extract physiological signals from the RGB-camera captured video [5]. Methods such as principal component analysis (PCA) and independent component analysis (ICA) have been used to decompose and recover HR signals from noisy video data [37]. However, these methods are limited by limitations such as head movement and ambient light intensity, which obscure the physiological signals being monitored in real-world conditions [39]. Additionally, demographic variations including skin tone can impact the reliability of rPPG signals which also reduces overall measurement accuracy [8]. Furthermore, HR measurements are impacted by different regions of interest (ROI) selection after face detection [40]. Recent study revealed that by focusing on larger skin areas such as the entire face can enhance rPPG signal-to-noise (SNR) ratio as well improve overall model performance [9].

Most existing signal-processing approaches require extensive fine-tuning to enhance overall signal quality which reduces the generalization while implementing rPPG method in real-life applications [16]. Moreover, many conventional methods cannot effectively handle the dynamic nature of environmental condition, as they rely on static models that cannot adapt to fluctuating light conditions and articulated motion artifacts (MAs) due to the subject’s movements [36]. As the quality of rPPG signals are highly influenced by these external factors, it requires the development of an advanced signal-filtering technique that can suppress noise from signal efficiently and improve overall HR measurement accuracy [12]. Additionally, as most available public datasets are recorded in a controlled environment, further studies must consider the acquisition of more diverse datasets under dynamic conditions [15].

To address these existing challenges, we obtained custom dataset that incorporates demographic variation and natural lighting conditions to simulate real-world settings. To ensure generalization of the proposed model, we also validated the framework on PURE dataset [49] where subjects are captured doing different activities such as quiet sitting, talking, and face rotation under indoor lighting. Further, we performed a comparative analyses on different ROIs on the subject’s face to evaluate model performance and error deviations on subjects with varying skin-tones. In this study, we proposed an adaptive signal-processing framework by integrating a wavelet-based denoising method (DWT) with residual-based adaptive Kalman filtering (RAKF), which outperforms conventional blind-source separation (BSS) techniques. The resulting DWT–RAKF integrated model enabled robust HR estimation by dynamically adjusting to MAs and ambient lighting through a signal-quality driven correction mechanism. Thus, the study offers the following contributions:

- Implementation of the DWT method to decompose raw RGB channels into multi-resolution components, followed by threshold-based denoising.
- Introduction of a novel RAKF method that dynamically updates its measurement noise covariance through frame-wise residuals, enhancing the model’s capability to suppress random fluctuations due to changes in motion and lighting conditions.
- Implementation of a signal-quality correction mechanism that is used to adaptively update the weight of the correction step of the proposed filter. This adaptive weighting enabled the system’s effective response to dynamic signal quality, thereby improving reliable measurement in uncontrolled environments.

The rest of the paper is organized as follows, review of related studies, overall materials and method section including experimental setup, data pre-processing stage, ROI detection, signal processing pipeline including signal denoising through wavelet decomposition, dual-stage bandpass filter, adaptive kalman filter, peak detection and HR measurement. In the result and discussion section, we evaluate the proposed method by comparing model performance metrics across diverse skin-tone variation and cross-dataset validation against the ground-truth measurements. Finally, this study concludes by summarizing findings and discussion on the future research directions.

## Related studies

In 2008 by utilizing cameras, Verkruysse et al. [2] first demonstrated that PPG signals exhibit varying intensity levels in RGB channels and that heart rate could be measured from facial video sequences. They revealed that HR signals could be reliably extracted from such video sequences by analyzing the intensity variations over time. Subsequently, Chen et al. [21] compared HR measurement accuracies across various skin regions, concluding that the facial skin provided the most reliable signal source for remote HR estimation.

In 2010, Poh et al. [4] introduced a blind-source separation (BSS) based noncontact cardiac pulse measurement method using color images. They computed the spatial average value of the facial region for each channel, where three temporal traces are obtained from an RGB video sequence. Independent component analysis (ICA) is applied to separate the observed raw signals and identify the underlying PPG signal. Finally, they applied fast Fourier transform (FFT) to the selected source signal to determine the highest power spectrum within the general human HR frequency band. Lewandowska et al. [7] proposed the principal component analysis (PCA) framework for rPPG signal extraction from raw traces and they evaluated the influences of different ROIs, color channel combinations, and ambient lighting conditions. Their findings confirmed that varying lighting conditions significantly impacted the accuracy of remote HR measurements.

In 2013, de Haan et al. [43] proposed CHROM method which models pulse information as a linear combination of RGB channels. It extracts the pulse signal by projecting normalized RGB channels onto chrominance components that are less affected by motion. However, it may struggle when pulse signals coincide with specular reflections [6]. Wang et al. [14] introduced the Plane-Orthogonal-to-Skin (POS) method for deriving the rPPG signal by projecting the RGB signal onto a plane orthogonal to the skin-tone vector to estimate HR. This approach performed reliably, particularly under challenging and varying lighting conditions. A follow-up study [9] showed that the POS method generated higher-quality pulse signals compared to CHROM [43], particularly under motion conditions.

In recent years, different studies introduced signal-processing algorithms for MA reduction and to improve the quality of rPPG signals [11,13]. Based on the blind source separation technique such as ICA method, few studies have proposed algorithm to obtain pulse-related components from rPPG signals. Similarly, Hui et al. [16] proposed a special ICA-based approach as joint approximate diagonalization eigenmatrices (JADE), where multiple enhancement filters are employed for frame normalization and lighting-variation reduction. Based on this approach, they performed FFT to determine the equivalent PPG signal in the frequency domain. Raghuram et al. [17] applied the Hilbert-Huang transform (HHT) to develop a decomposition technique that suppressed MA in rPPG signals. This algorithm exploited the empirical mode decomposition of nonlinear data to obtain the intrinsic-mode function (IMF) elements as well as analyze the changes in amplitude over time. Other notable approaches include the integration of least mean squares filtering with singular value decomposition and the slope sum method (SSM) [18,33]. These approaches are tested against the tunable Q-factor wavelet transform, where they remarkably suppressed motion artifacts. Similarly, a time–frequency domain approach employing two adaptive filters in sequence alongside singular spectrum analysis (SSA) is proposed for spectral peak enhancement [34]. Another study conducted a comparative analysis of two techniques for reducing MAs in rPPG signals by including the wavelet-transformation approach and the dynamic signal filtering approach. Their findings indicated that wavelet-based reconstruction is effective, particularly for HR and pulse transit time analysis [32]. In 2018, Prakash et al. [36] proposed bounded Kalman filtering for motion estimation and feature tracking during noncontact HR measurement.

Overall, this review reveals that most conventional camera-imaging based signal processing approaches in noncontact HR estimation applied different signal filtering strategies and BSS techniques to improve overall signal quality. Although widely used as a benchmark for new developments, the BSS technique is sensitive to facial movements, ambient light intensity and skin-tone variations [38]. These environmental noise can only be mitigated through extensive post-processing and manual fine-tuning of rPPG signals, limiting the real-time application of conventional approaches and requires the development of novel method that can handle these challenges effectively [15].

## Materials and methods

In this study, we proposed an advanced signal processing framework for rPPG-based noncontact HR estimation. This framework integrates residual-based adaptive Kalman filter (RAKF) and multichannel signal fusion based on dynamic weighting for temporal smoothing and spatial feature extraction. The proposed algorithm dynamically adjusts the measurement noise covariance based on the residual, thereby ensuring robust HR estimation under low SNR conditions and intensities. Additionally, we integrated discrete wavelet transform (DWT) for signal denoising and dual-stage bandpass filtering techniques to estimate HR by adapting the light intensity and overall signal quality enhancement to remove noise and MAs.

**Fig 1:**
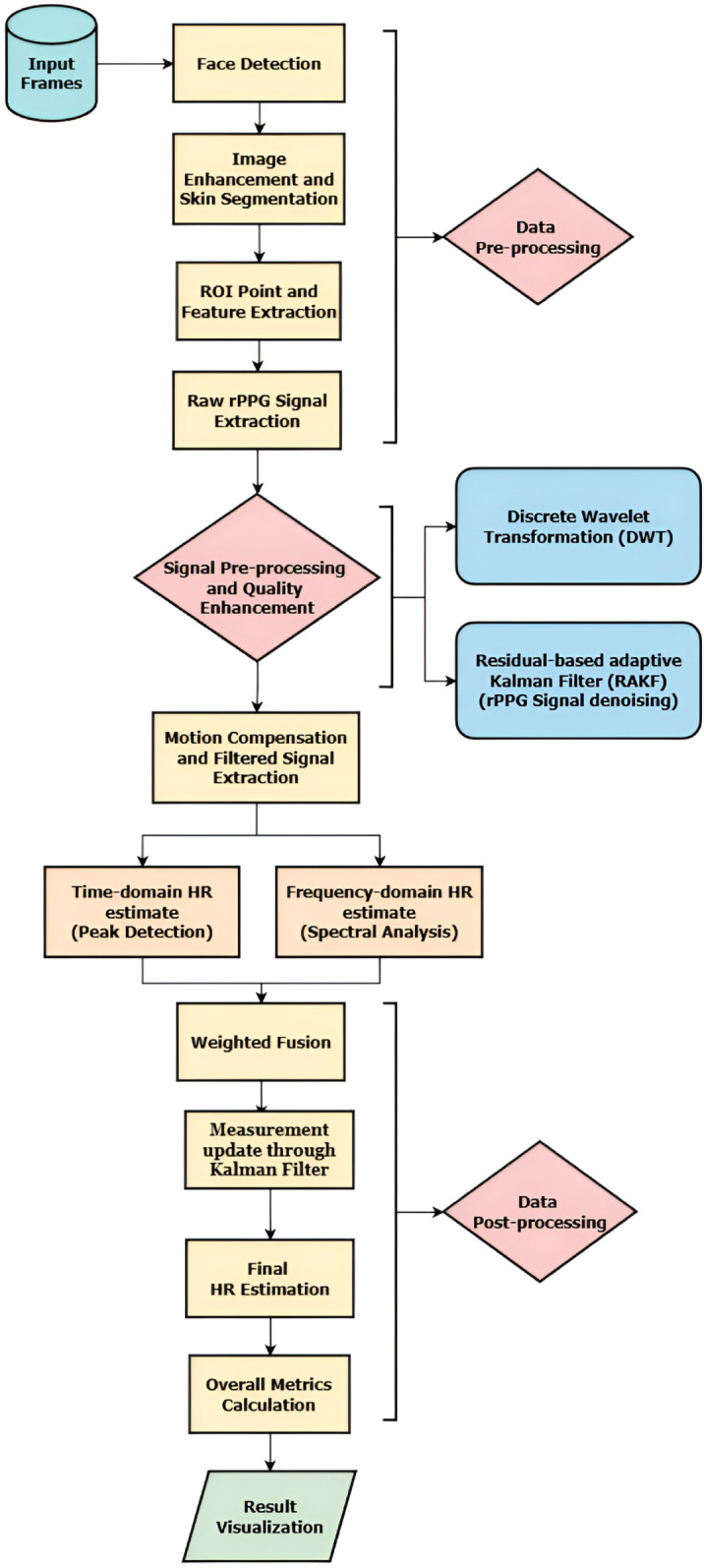
Overview of the proposed methodology.

This adaptive filtering enabled effective HR estimation from input frames by outperforming the conventional signal filtering and BSS techniques. Fig 1 shows overall workflow of the proposed algorithm including each stages.

### Experimental setup and data collection

**Fig 2:**
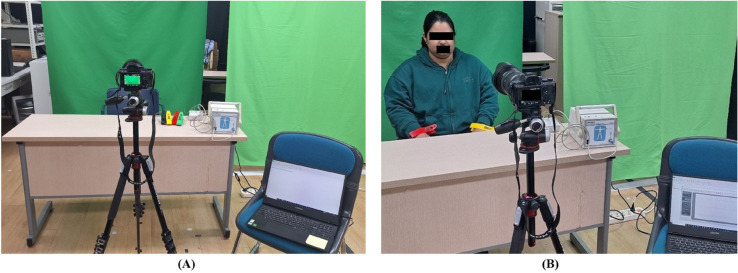
Experimental setup and dataset collection. (A) Overview of the experimental setup, (B) Custom database collection while the subject sitting from 1 m away from camera and corresponding reference HR measurement are performed an using QECG sensor.

We collected custom datasets, including those of subjects with demographic variations, mostly distributed around South and East Asia. To validate our proposed approach, we evaluated the framework on both public and custom datasets. The experimental data are collected using a Sony Alpha 7 III camera, which recorded videos at resolutions of 6000 x 4000 pixels. To maintain the consistency of the images and framing rate of the captured video, the camera is set at a fixed distance of 1m from the subjects as shown in Fig 2(A). The videos are recorded at 30 fps under ambient lighting conditions, characterized by natural light variation comprising shaded and lit regions. The subjects are sitting still in front of a green background, and the other environmental conditions are kept constant. Participants are instructed to remain seated and take normal breaths, and no further measures are adopted to minimize noise or disturbance during the recording. The obtained dataset included the recordings of ten subjects (five males and 5 females), specifically South and East Asians aged between 25 and 35 years old, with varying skin colors to ensure broad system validation. The participants provided written informed consent delivered through detailed consent forms, and the experiment is conducted in one day February 11, 2025. Further, we obtained the ethical approval from the Yeungnam University Institutional Review Board (Approval number: 7002016-A-2024-114), and data are accessed and analyzed between February 12 and April 30, 2025 to develop and validate the algorithm. Each video recording session is conducted for 60 seconds. To capture the ground truth, reference heart rate is simultaneously recorded using an ECG sensor as shown in Fig 2(B), allowing for the accurate validation of the contactless measurements. The corresponding ground-truth data are recorded in an .xlsx file for subsequent comparative analysis.

### Data pre-processing and ROI detection

**Fig 3:**
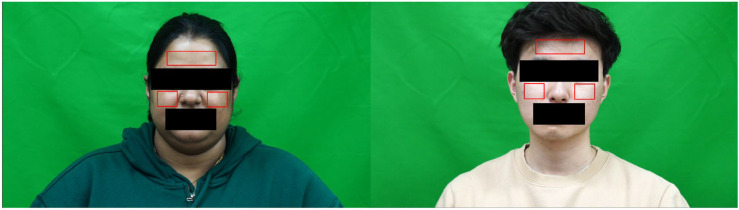
Overview of ROI selection from detected facial regions(forehead and cheeks). Subjects exhibited skin tone variations.

**Fig 4:**
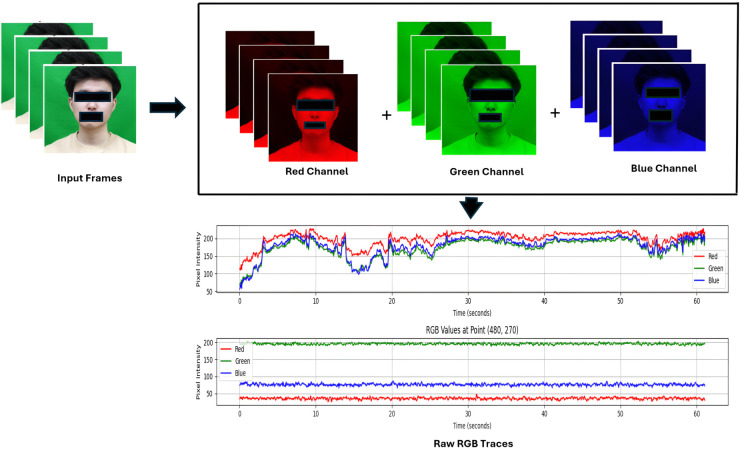
Overview of raw signal extraction through RGB channel fusion.

First, the input video is standardized to ensure consistency and improve quality across all the recordings. As a previous study [35] revealed that enhanced resolutions yielded improved HR estimation results, we maintained the high-pixel densities to ensure accurate measurements under diverse conditions. To manage low-light conditions, the system assessed the frame brightness and improved image quality through a contrast enhancement approach (CLAHE). The system transformed each video frame into grayscale images. Following the initial frame enhancement, the system employed a Haar feature-based cascade classifier [27] to detect the subject’s faces. Once the face is reliably detected, three key ROIs are dynamically determined as forehead, left and right cheeks. After that, the detected ROIs are divided into multiple smaller sub-ROIs to reduce the impact of uneven illumination and facial expression. Fig 3 gives an overview of ROI selection and Fig 4 presents an overview of the extraction of raw signal through the integration of multiple RGB channels.

### Color channel extraction

**Fig 5:**
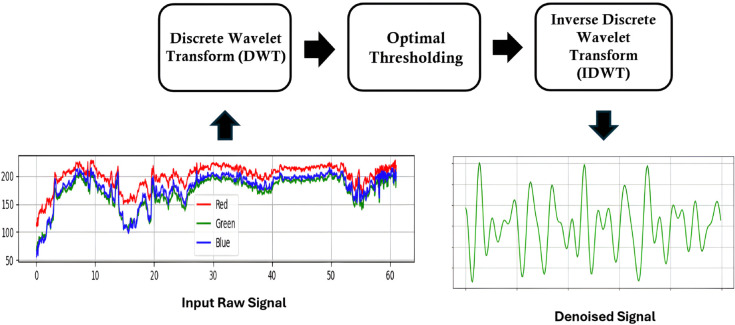
Overview of the discrete wavelet transformation (DWT) method.

From each ROI, we computed the average pixel intensity over time for the green channel, which is most sensitive to hemoglobin absorption. Let $\text{ROI}\subset {\mathbb{R}}^{2}$ be the selected ROI (e.g. forehead and cheeks) within a detected face where *I*_*c*_(*i*,*j*,*t*) denote the pixel intensity at spatial coordinates (*i*, *j*) and time *t* for a given color channel, c∈{r,g,b}. The temporal color signal *g*(*t*) is computed as the spatial average of the pixel intensities within the ROI:

$$g(t)=\frac{1}{N}\sum_{(i,j)\in \text{ROI}}I_{c}(i,j,t)$$ (1)

where, N is the total pixel number in the selected ROI. We repeated this process for each frame *t* and color channel *c* and the resulting time-series signal *g*(*t*), served as the raw PPG signal. Fig 5 gives an overview of the raw signal extraction process.

### Signal denoising through discrete wavelet transform (DWT)

To suppress noise from the raw PPG signal *g*(*t*), we employed wavelet-based denoising using the discrete wavelet transform (DWT). In this framework, the signal is represented as a linear combination of scaled and shifted wavelet basis functions:

$$g(t)=\sum_{j=0}^{J}\sum_{k}c_{j,k}\;\psi_{j,k}(t)$$ (2)

where *c*_*j*,*k*_ are the wavelet coefficient at scale *j*, *k*, $\psi_{j,k}(t)$ are the wavelet basis function, and *J* is the maximum decomposition level. Here the indices *j* and *k* play distinct role in decomposition. The scale index *j* corresponds to the decomposition level, with lower values of *j* captures high frequency details which are associated with noise whereas higher values captures lower frequency corresponding slowly varying trends. The translation index *k* determines the position of the wavelet in time, enabling localization of transient noise or features. The parameter *J* denotes the maximum decomposition level, effectively setting the depth of analysis and the finest resolution available for separating noise from meaningful signal structure. In wavelet-based denoising, the suppression of small wavelet coefficients is often referred to as shrinking. In our implementation, the coefficient suppression is performed using a data-driven threshold selection rule. Specifically, the threshold value *T* is derived from the noise variance using the median absolute deviation (MAD) of the finest-scale detail coefficients. Following Donoho and Johnstone’s universal thresholding rule [42], the threshold value is defined as:

$$T=\sigma \sqrt{2\log n}$$ (3)

where, *n* is the signal length and *σ* is the median absolute deviation of the detail coefficients at the first decomposition level. This data-driven procedure is considered as optimal as it minimizes the mean squared error of signal reconstruction under Gaussian noise conditions. So for denoising, wavelet coefficients at each level are subjected to optimal thresholding:

$${\tilde{d}}_{j,k}=\left\{ \begin{array}{cc} \text{sgn}(d_{j,k})\cdot (|d_{j,k}|-T), & \text{if }|d_{j,k}|>T\\ 0, & \text{otherwise} \end{array}\right.$$ (4)

where *d*_*j*,*k*_ are detail coefficients at scale *j* and threshold *T*. This process selectively attenuates small coefficients like noise while preserving significant oscillations associated with physiological signal dynamics. Finally, the denoised signal $\hat{g}(t)$ is reconstructed using the inverse wavelet transform as:

$$\hat{g}(t)=\sum_{j=0}^{J}\sum_{k}{\tilde{c}}_{j,k}\;\psi_{j,k}(t)$$ (5)

Fig 5 gives an overview of the wavelet-denoising process for suppressing noise from the raw signal.

### Dual-stage bandpass filtering

To further refine the signal, we adopted a dual-stage filtering strategy as follows:

#### Stage 1: Wide bandpass filter.

The preliminary wide bandpass (0.6–3.5 Hz) is implemented by using Butterworth bandpass filter [57] to attenuate low-frequency baseline wander such as head motion, illumination changes, slow drifts, and high-frequency sensor noise. By removing these noise artifacts, this stage produce a cleaner signal for time-domain analysis. Previous studies have demonstrated that rPPG signals are highly susceptible to low illumination changes and motion-induced fluctuations which should be removed to prevent pulse distortion and spectral leakage [54–56]. Therefore, the wide bandpass filter ensures that subsequent processing starts from a refined signal that is confined to the broad physiological range of cardiac activity while maintaining sufficient spectral information. It improves the signal quality by removing slow baseline drift and produces more stable signal for spectral analysis in the following stage.

#### Stage 2: Narrow bandpass filter.

In the second stage, narrow bandpass filter (0.8–2.5 Hz) focuses specifically on the frequency range where the HR is most dominant. Narrowing the passband at this stage improves robustness of the signal against motion harmonics by eliminating unwanted components such as MAs and ambient noise. The following equation comprehensively explains the proposed pipeline, where *H*(*f*) is the frequency response of the bandpass filter, and *f*_*L*_ and *f*_*H*_ are the lower and upper cutoff frequencies.

$$H(f)=\left\{ \begin{array}{cc} 1, & \text{if }f_{L}\leq f\leq f_{H}\\ 0, & \text{otherwise} \end{array}\right.$$ (6)

The filtered signal is computed as follows:

$$\hat{s}(t)=\hat{g}(t)*h(t)$$ (7)

where, *h*(*t*) is the impulse response of the filter. Following the signal enhancement through wavelet denoising and multi-bandpass filtering, an initial pulse-related frequency component is observed through spectral analysis. Specifically, the preprocessed rPPG signal $\hat{s}(t)$ is analyzed in the frequency domain through FFT:

$$S(f)=ℱ\{\hat{s}(t)\}$$ (8)

where, *S*(*f*) represents the frequency spectrum of the signal. The dominant spectral component is identified within a predefined physiological frequency range, and the initial observation *z*_*k*_, is calculated. The subsequent RAKF stage leveraged these frame-wise initial estimates to predict temporally consistent HRs by optimally balancing between the model prediction and measurement correction.

### Residual-based adaptive Kalman filtering

The Kalman filter is a recursive Bayesian estimator for the hidden state *x*_*k*_ from noisy measurements, where *z*_*k*_ is the observed pulse rate at time frame *k* from the filtered rPPG signal calculated through spectral analysis. Owing to MA, lighting changes, and video compression the signal might exhibit high variance and abrupt fluctuations. To refine this noise, we modeled the reference HR, *x*_*k*_  as a hidden state evolving according to a random walk model. For $x_{k},z_{k}\in \mathbb{R}$:

$$x_{k}=x_{k-1}+w_{k-1},\;w_{k-1}\sim 𝒩(0,Q_{k})\;z_{k}=x_{k}+v_{k},\;v_{k}\sim 𝒩(0,R_{k})$$ (9)

where $w_{k-1},v_{k}$ is the independent white Gaussian noise. Here, *Q*_*k*_ and *R*_*k*_ are the process noise and the measurement noise covariances.

#### Prediction step:

This phase is considered the prediction phase of the Kalman filter, where ${\hat{x}}_{k}^{-}$ is the predicted state or prior estimate, and $P_{k}^{-}$ is the predicted error covariance.

$${\hat{x}}_{k}^{-}={\hat{x}}_{k-1}\;P_{k}^{-}=P_{k-1}+Q_{k}$$ (10)

#### Residual step computation:

The residual, denoted by ${\tilde{y}}_{k}$ is defined as the difference between the actual observation and predicted measurement, ${\hat{x}}_{k}^{-}$, obtained in the prediction phase of the Kalman filtering:

$${\tilde{y}}_{k}=z_{k}-{\hat{x}}_{k}^{-}$$ (11)

The residual is used to update the predicted state in a manner that minimizes the posterior estimation error covariance.

#### Adaptive measurement noise update:

In classical Kalman filtering, the measurement noise covariance, $R_{k}$ is typically assumed to be constant. However, under realistic conditions, the reliability of the observed measurement, $z_{k}$, may vary significantly over time owing to factors such as sensor noise and lighting variations. To address this, we define an adaptive measurement-noise covariance as follows:

$$R_{k}^{\text{adaptive}}=R_{0}\left(1+\frac{\left|{\tilde{y}}_{k}\right|}{\beta }\right)$$ (12)

where, *R*_0_ is the basal measurement noise covariance and β> 0 is the sensitivity parameter. This step increases capability of the filter to reject unrelated frequencies from noise artifact-induced physiological signals.

#### Kalman gain computation:

The proposed Kalman filter is used to compute a Kalman gain $K_{k}$, to control the extent to which the new measurement influenced the final estimate serving as a weighting factor that determines the relative contribution of the predicted state and new measurement to the updated estimate.

$$K_{k}=\frac{P_{k}^{-}}{P_{k}^{-}+R_{k}^{\text{adaptive}}}$$ (13)

$P_{k}^{-}$ is the a priori estimation-error covariance and $R_{k}^{\text{adaptive}}$ is the adaptive measurement noise covariance. High signal quality corresponds to enhanced gain and an increased residual value corresponds to decreased Kalman gain indicating potentially unreliable measurement.

#### Outlier handling using residual-informed observation correction:

To prevent the corruption of the state update of the filter by outlier measurements, a residual-informed outlier handling strategy is introduced to compare the current observation with a robust local reference as follows:

$$\text{if }|z_{k}-{\bar{z}}_{k}|>T_{\text{out}},\;\text{then }z_{k}\leftarrow {\bar{z}}_{k}$$ (14)

where, ${\bar{z}}_{k}$ is the median of *N* previous observations and $T_{\text{out}}$ is the predefined threshold value. This ensures that abrupt motion artifacts, illumination fluctuations do not bias the state update or degrade the accuracy of the filter’s state estimates.

#### State update with adaptive weighting:

In the proposed framework, the update of the state estimate is weighted additionally based on the signal quality index (SQ) of the rPPG signal. While the adaptive measurement noise-covariance $R_{k}^{\text{adaptive}}$ adjusts the Kalman gain *K*_*k*_ as per residual statistics, this adaptation reflects the measurement reliability. However, in realistic scenarios, signal quality can degrade due to motion, illuminations changes and compression artifacts which may not be fully be captured by residual-based noise adaption [44]. To address this issue, we introduced a dynamic weighting factor $w_{k}$ as:

$$w_{k}=\max(\alpha ,{\text{SQ}}_{k})$$ (15)

where, ${\text{SQ}}_{k}\in [0,1]$ is the signal-quality index and *α* is a minimum weight threshold ensures the update contribution in cases of poor signal quality. The Signal quality index (SQ) is computed by combining spectral and temporal features, including the spectral power ratio (SPR), signal-to-noise ratio (SNR), and the peak stability (PS) of inter-beat intervals over a local window:

$$SQ_{k}=\lambda_{1}\cdot SPR+\lambda_{2}\cdot SNR+\lambda_{3}\cdot PS$$ (16)

Here, the weighting parameter $\lambda_{j}$ are empirically selected for j=1,2,3 where $\lambda_{1}$ = 0.3, $\lambda_{2}$ = 0.4, $\lambda_{3}$ = 0.3. SNR values are assigned with higher weights because frequency-domain characteristics are more discriminative for assessing the reliability of quality of the signal [59]. Here, the constraint $\lambda_{1}+\lambda_{2}+\lambda_{3}=1$ ensures the *SQ*_*k*_ values are normalized and bounded between 0 and 1 at time step *k*. Higher *SQ*_*k*_ values indicate more reliable rPPG signals. After computing Kalman gain and dynamic weighting factor, the proposed filter updated the predicted state, ${\hat{x}}_{k}$, to estimate the new posterior estimate and the update equation as follows:

$${\hat{x}}_{k}={\hat{x}}_{k}^{-}+K_{k}\cdot w_{k}\cdot (z_{k}-{\hat{x}}_{k}^{-})$$ (17)

Here, *w*_*k*_ moderates the correction term by reducing the impact of low quality observations and preserving the recursive update process. After updating the state, we updated the error covariance, *P*_*k*_ where *w*_*k*_ is not included to preserve the statistical consistency of the filter. The covariance update is defined as

$$P_{k}=(1-K_{k})P_{k}^{-}$$ (18)

This ensures that the error covariance continues to reflect the probabilistic uncertainty propagation based solely on the system and measurement noise definitions. This recursive update improved overall signal quality by mitigating the frame-level noise and rejecting the transient outliers. Under Gaussian-noise conditions, the filter provided linear unbiased estimate of the hidden state by integrating the prior state predictions with updated state observations. Thus, proposed Kalman filter serves as an optimal estimator that can provide stable real-time HR tracking in dynamic and uncontrolled environments. Additionally, to evaluate efficiency of the proposed method, we compared the runtime complexity of our proposed algorithm with conventional signal processing approaches. Proposed algorithm is executed on a standard CPU with intel Core i7 processor using Python. As shown in the result section, proposed signal processing framework outperformed existing SOTA models with lowest inference time while enabling reliable HR measurement using video recordings under challenging conditions including varying illumination, motion artifacts, and diverse subjects.

## Peak detection and HR measurement

Next, HR is estimated from the filtered rPPG signal, $\hat{s}(t)$ following the signal-processing framework. The pulse-related component is computed by identifying the temporal positions of successive systolic peaks in $\hat{s}(t)$. Let $\left\{t_{1},t_{2},\ldots ,t_{N}\right\}$ denote the time stamps of the *N* detected peaks over a signal duration of *T*. The inter-peak intervals are defined as follows:

$$\Delta t_{i}=t_{i+1}-t_{i},\;\text{For }i=1,\ldots ,N-1$$ (19)

The average inter-beat interval (IBI) is given as follows:

$$\overset{―}{\Delta t}=\frac{1}{N-1}\sum_{i=1}^{N-1}\Delta t_{i}$$ (20)

The peak detection algorithm is implemented within the relevant HR frequency range of 0.75–0.25 Hz (45–150 bpm), where the peaks are defined based on the accepted conventions with regard to distance, prominence, and width. Finally, HR is determined by multiplying the peak frequency by 60.

$$f_{\text{peak}}=\frac{1}{\overset{―}{\Delta t}}$$ (21)

$$\text{HR (BPM) }=60\times f_{\text{peak}}$$ (22)

To obtain consistent HR estimates, peak detection is first applied to the denoised rPPG signal $\hat{s}(t)$, which reduces the influence of random noise and motion-induced fluctuations. In addition, a spectral-domain HR estimate is computed. These two complementary estimates from time-domain and spectral-domain are then combined using a weighted fusion scheme. The fusion weights are determined by the corresponding signal quality indices using signal quality index (SQ). The estimate with the higher signal quality is assigned larger weight, ensuring that the more reliable source contributed to the final HR estimation. Subsequently, the weighted fusion scheme maintained consistency across frame-by-frame feature extraction and realizing more highly precise HR estimation over a given period.

## Results and discussion

Here, we discuss the performance of the proposed model, which has been evaluated using PURE and custom dataset. To evaluate the results, we employed the following performance evaluation metrics as mean absolute error (MAE), root mean square error (RMSE), and signal-to-noise ratio (SNR). MAE and RMSE value indicates the accuracy of the proposed model for HR estimation, while the SNR value indicates the effectiveness of the proposed filter for noise suppression and motion artifact removal in the rPPG signal.

### Performance evaluation metrics

Here, MAE and RMSE metrics offer insights into the error magnitude between the estimated HR and the ground-truth ECG reference value. The metrics are defined as follows:

$$\text{MAE}=\frac{1}{N}\sum_{i=1}^{N}|y_{i}-{\hat{y}}_{i}|,$$ (23)

$$\text{RMSE}=\sqrt{\frac{1}{N}\sum_{i=1}^{N}(y_{i}-{\hat{y}}_{i}{)}^{2}}.$$ (24)

where, the predicted HR values are ${\hat{y}}_{i}$ and the ground-truth values are *y*_*i*_ over *N* number of samples.

### Signal quality assessment (SNR)

To quantify rPPG signal quality after denoising, we computed the signal-to-noise ratio (SNR) value. Assuming the processed signal $\hat{s}(t)$ is mean-centered, the signal power $P_{\text{signal}}$ is defined as:

$$P_{\text{signal}}=\frac{1}{N}\sum_{i=1}^{N}{\hat{s}}^{2}(t_{i}).$$ (25)

Noise is estimated by computing the deviation between the raw rPPG signal *g*(*t*) and the denoised signal $\hat{s}(t)$. The noise component, *n*(*t*) is given as follows:

$$n(t)=g(t)-\hat{s}(t).$$ (26)

Accordingly, the noise power $P_{\text{noise}}$ is defined as:

$$P_{\text{noise}}=\frac{1}{N}\sum_{i=1}^{N}n^{2}(t_{i}).$$ (27)

Next, SNR is computed as the ratio of the signal-to noise-powers. To express in decibels(dB), the SNR value is computed as follows:

$${\text{SNR}}_{\text{dB}}=10\log_{10}\left(\frac{P_{\text{signal}}}{P_{\text{noise}}}\right).$$ (28)

**Fig 6:**
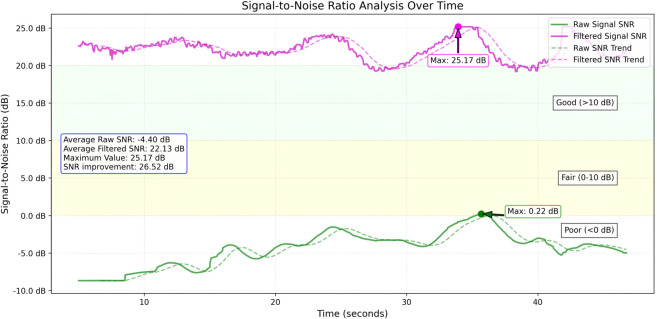
SNR analysis. This plot shows the SNR values of filtered vs.raw signals over time.

Fig 6 shows the SNR analysis over time to compare the raw and filtered signal qualities. The raw signal exhibited consistently low SNR levels, with an average of –4.40 dB, remaining below the threshold value and in the range of poor signal quality. In contrast, the filtered signal demonstrated substantial enhancement in its signal quality achieving an average of 22.13 dB corresponding to an overall SNR improvement of 26.52 dB compared with the raw signal. Despite the significant noise present in initial 5–10 s interval, the proposed method effectively preserved the temporal dynamics of the rPPG signal while improving the overall signal quality to predict the HR.

### Sensitivity analysis

To ensure a dataset-independent hyperparameter selection, we performed a sensitivity analysis across a range of values for each parameter. In our proposed method, each hyperparameter are set based on physiologically relevant HR ranges and signal characteristics rather than dataset-specific tuning. The maximum wavelet decomposition level *J* is chosen so that DWT filter captured the HR frequency band. The threshold value *T* in Eq (4) followed the universal thresholding rule where, *σ* is median absolute deviation of the detailed coefficients at the decomposition level. In Eq (15), basal measurement noise covariance *R* and β is selected to ensure robustness against transient fluctuations. As Kalman filter parameters are set based on specific applications [58], basal measurement noise covariance *R*_0_ and process noise covariance *Q*_*k*_ values are set as fixed values across all datasets. Both *Q*_*k*_ and *R*_0_ are empirically selected to balance filter tracking responsiveness with measurement noise suppression. The basal measurement noise covariance is fixed to *R*_0_= 25 bpm and process noise covariance *Q*_*k*_ = 2 × 10^−4^ as it provides optimal performance across different illumination and motion scenarios while maintaining temporal stability. In Eq (17), the outlier rejection threshold $T_{\text{out}}$ is set based on standard deviations of median-filtered residuals and the weight threshold value *α* is selected to update contributions in cases of poor signal quality from each frames.

**Fig 7:**
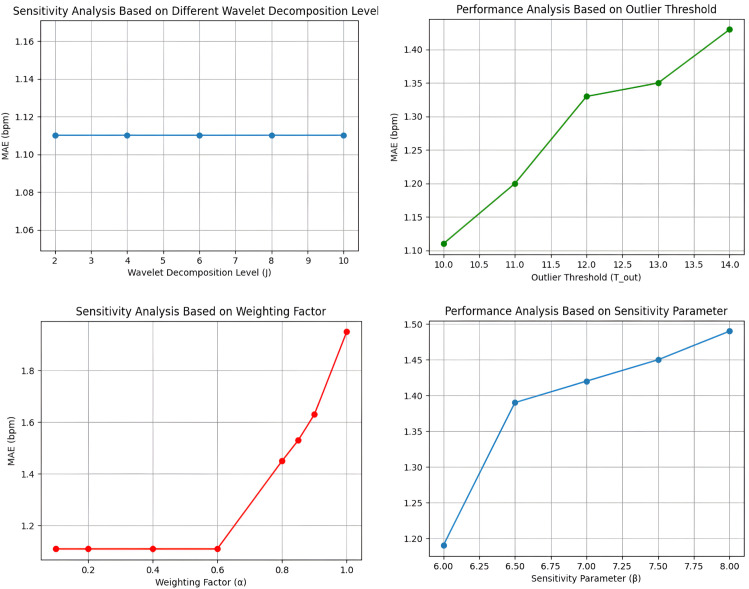
Sensitivity analysis. This plot shows the sensitivity analysis of the proposed method based on different hyperparameter values.

Fig 7 shows the sensitivity analysis of the proposed model based on different hyperparameter choices and overall performance metrics. As shown in Fig 7, across different wavelet decomposition levels (*J*), overall mean average error (MAE) remains constant. This consistency occurs because the DWT stage primarily functions as a noise suppression mechanism while effectively attenuating high-frequency noise components. For the weighting factor (α), the performance remains stable between 0.1 to 0.6 but model performance degrades progressively beyond α= 0.7. It indicates that the quality-based frame differentiation is essential for achieving higher model performance. For different sensitivity parameter (β) and outlier rejection threshold ($T_{\text{out}}$), some slight variations have been observed in performance metrics but the effect is marginal and below < 0.3 bpm. These findings suggest that the overall model performance metrics is not dependent on dataset-specific hyperparameter tuning and shows strong generalization while predicting HR accurately.

### Dataset-specific performance analysis

First, we analyzed the performance of the proposed model on the PURE dataset. As presented in Table 1, the proposed method obtained MAE and RMSE scores of 0.72 and 1.14 bpm respectively, which are compared with those of the ground-truth reference. To improve model generalization, we tested our model on the custom dataset comprising video recordings of the subjects with skin-tone variations under natural-lighting conditions. We observed that our proposed signal-processing denoised and suppressed noise efficiently while estimating HRs from the fair-skinned tone subjects with an MAE of 0.94 bpm. In contrast, it obtained a slightly higher MAE value for the dark-skinned subjects (1.11 bpm). Table 1 presents the details of the overall comparative analysis of the performance of the proposed model against those of different state-of-the-art (SOTA) models for HR measurement using PURE and custom datasets. To assess time complexity the proposed model, we compared inference delay of the proposed framework execution with conventional signal processing approaches. As shown in Table 1, proposed framework outperformed existing modles with an average inference time of 7.6 ms per frame while testing on PURE dataset. Additionally, an ablation study has been conducted of the proposed framework. As presented in Table 2, the ablation study indicates that though DWT and RAKF showed effective noise suppression, they show poor performance handling of spatial variability and dynamic artifacts present in uncontrolled environments—limiting their performances when the are implemented separately. Therefore, we integrated them with a signal-quality-aware signal-processing strategy to ensure robust rPPG signal extraction and improve model performance across diverse datasets.

**Table 1:** Performances of different HR measurement methods on public and custom datasets.

Method	Dataset	MAE (bpm)	RMSE (bpm)	Inference time (ms)
Green [2]	PURE	10.14	23.86	-
ICA [6]	PURE	3.91	12.61	31
CHROM [10]	PURE	5.73	14.92	28
POS [14]	PURE	3.63	11.82	27
ProjectICA [20]	PURE	2.56	8.05	-
EEMD+FastICA [29]	PURE	2.87	7.61	-
2SR [28]	PURE	2.44	3.06	-
Self-Adaptive SSA [22]	PURE	2.05	5.27	48.3
Comas et al. [23]	PURE	1.83	2.30	-
MCS [24]	PURE	1.65	2.90	-
Gideon et al. [19]	PURE	2.30	2.90	-
HR-CNN [25]	PURE	1.84	2.37	-
EfficientPhys [31]	PURE	1.14	1.81	40
Cpulse [30]	PURE	0.98	1.94	-
Var-HR [26]	PURE	0.84	1.33	-
Proposed method	PURE	**0.72**	**1.14**	**7.6**
Proposed method	Custom (Dark)	**1.11**	**1.46**	**14.2**
Proposed method	Custom (Light)	**0.94**	**1.38**	**15.8**

**Table 2:** Ablation study through intra-dataset testing.

Method	PURE (Public)	Custom
DWT	12.75	10.94
RAKF	2.87	3.36
RAKF + Dynamic weighting	1.95	2.10
DWT + RAKF (Without dynamic weighting)	0.85	1.96
DWT + RAKF (Proposed framework)	**0.72**	**1.11**

**Notes:** “DWT” indicates discrete wavelet transformation method without adaptive Kalman filter. “RAKF” indicates only adaptive Kalman filtering without discrete wavelet transformation method. “RAKF + Dynamic weighting” indicates integration of weighting mechanism with adaptive Kalman filter. “DWT + RAKF” indicates full implementation of the proposed framework for filtering and denoising signal. All reported values (MAE in bpm) are evaluated on both public and custom dataset.

**Fig 8:**
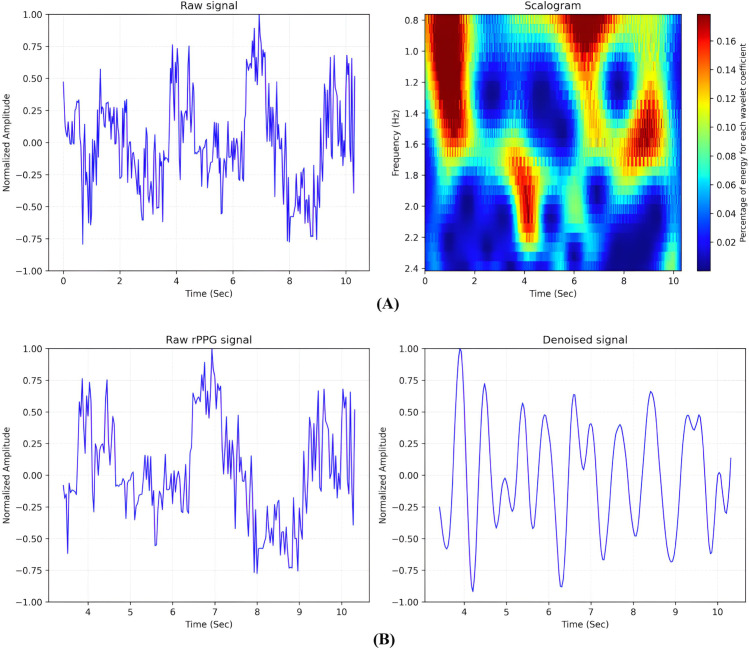
Scalogram plot. (A) Raw Signal with Corresponding Scalogram (B) Noisy rPPG Signal and Denoised Signal. This plot shows the sensitivity analysis of the proposed method based on different hyperparameter values.

Fig 8 shows Scalogram analysis of the raw signal obtained using continuous wavelet transformation (CWT) which shows percentage of energy levels for each wavelet coefficients, guiding the selection of decomposition levels for the subsequent DWT-based filtering. As shown in the Fig 8(A), the raw signal extracted from the input frames over a 10s window exhibited significant noise, making it difficult to distinguish physiological components such as heartbeats. The scalogram decomposed this signal into frequency components revealing which frequencies dominated at specific times. It also revealed the time-frequency content of the signal, while the energy concentrated in the frequency range of 0.8 to 2.4 Hz, corresponding to the pulse-related component. As shown in the color map, the power density of the signal where the red and yellow regions exhibited high energy as well as the strong presence of the related frequency components and the blue region exhibited low signal energy related to the noise. Fig 8(B) indicates the detrended rPPG signal and its temporal dynamics after applying the discrete wavelet-based denoising method filtering out the unrelated signal components. The bottom-right subplot shows the denoised signal obtained through adaptive filtering exhibiting a smooth, quasi sinusoidal physiological waveform corresponding to HR.

**Fig 9:**
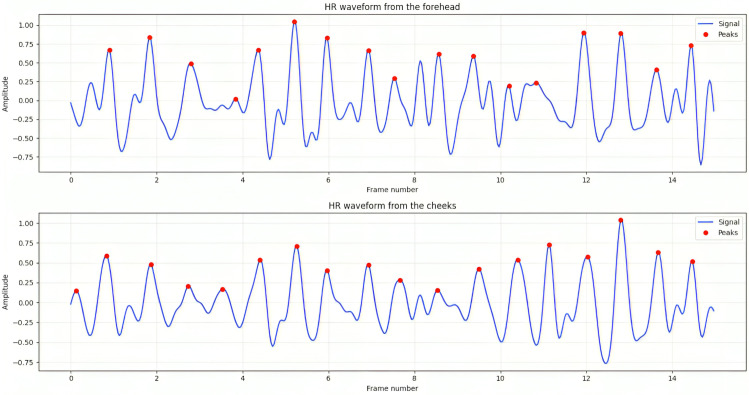
Peak detection of from the HR waveform. This plot shows the detected peak and corresponding HR cycle from both ROIs.

As mentioned in the previous section, our proposed approach explored the forehead and cheek regions as the key ROIs from the facial region and analyzed the performance of the model for each region to find which ROI facilitated relatively enhanced performance. From the result analysis as shown in (Fig 9), although both regions showed marginally compatible performances, the cheek regions contained more pulsatile information and ensured higher accuracy and lower error metrics.

**Fig 10:**
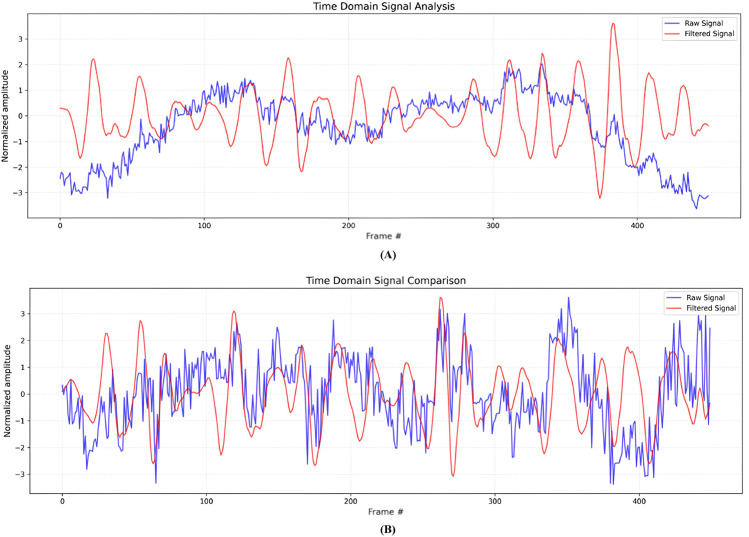
Time-domain analysis (A) PURE and, (B) Custom datasets. This plot shows the adaptive filtering framework’s ability to remove noise from the raw signal and restore the pulse physiological waveform in the time domain.

**Fig 11:**
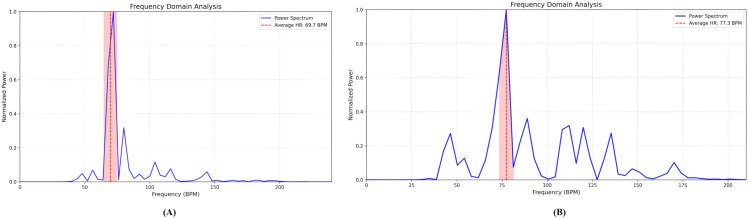
Frequency-domain analysis (A) PURE and, (B) Custom datasets. This plot shows the PSD indicating the dominant peak after frequency-domain analysis.

To further analyze the performance of the proposed model, we conducted a time-domain analysis between the raw and filtered signals during post and prior signal-processing to evaluate the effectiveness of the applied method. As shown in Fig 10, MAs and ambient lighting changes caused the raw signal to contain substantial noise. However, employing the proposed filtering algorithm, we removed MAs from the raw signal and extracted HR-related signal efficiently. The power spectral density (PSD) graph is calculated using Welch’s method [41]. As shown in Fig 11, it exhibited the dominant peak corresponding to HR frequency components within the frequency band from the filtered signal.

**Fig 12:**
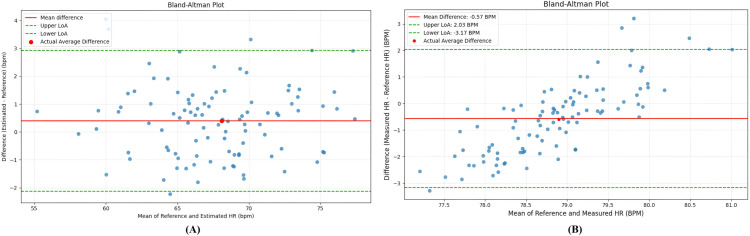
Bland-Altman plot. (A) Subject with dark brown skin tone (on Fitzpatrick scale type V), (B) Subject with fair skin tone (on Fitzpatrick scale type II).

To evaluate the agreement between the proposed HR estimation method and the ground-truth measurement, the Bland–Altman plots are generated separately (Fig 12) for the subjects with different skin-tones. For the dark-skin group (Fig 12)(A), slightly higher differences are observed between the measured and reference HR, indicating slight overestimation on average owing to less light reflection because of the melanin concentration. As shown in the figure, most of the points are close to the mean average, with limits of agreement (LoA) ranging from –1.95 to +2.75 BPM. A narrow LoA band and uniform distribution of predicted values around mean zero line indicate a high level of agreement and minor systematic bias across the predicted HR values between frames. On the fair-skin group subjects, the proposed algorithm revealed a lower mean difference, indicating a slight underestimation. As shown in the Fig 12(B), the LoA band drew closer as the residuals remained evenly distributed, indicating consistent performance across both skin-tones. The result analysis demonstrates that the proposed adaptive signal-filtering framework can reliably estimate the HR by maintaining frame-by-frame temporal consistency and improving the signal quality.

In controlled environments as shown in the PURE dataset, proposed method performed well, resulting in higher accuracy and lower error than the existing SOTA models. However, in more real-world scenarios, the overall accuracy slightly reduces owing to factors like varying lighting conditions, subject movement, and differences in skin reflectance. We also noticed that differences between individuals with lighter and darker skin tones made a difference in the model’s performance. Specifically, darker skin, with higher melanin content, tends to reflect less light from the skin area. It reduces the overall signal quality due to the low SNR and increases the measurement errors. Despite these challenges, the proposed method demonstrates strong generalization and adaptability through its signal quality-aware weighting and real-time filtering strategy. These findings highlight the potential of the proposed method for real-time implementation in a camera-based physiological monitoring system across diverse demographics.

## Conclusion

In this study, we introduced an adaptive signal processing framework for the vital sign measurement using the rPPG method which can accurately measure HRs irrespective of the lighting condition and skin tone variation. The proposed algorithm integrates discrete wavelet-based denoising and residual-based adaptive Kalman filter (RAKF) to enhance temporal consistency and improve signal quality by reducing the measurement noise. A signal quality index is used to adaptively weight the proposed filter’s correction step, enabling robust performance across varying lighting conditions. Overall, this study introduces an advanced signal processing framework that can improve the signal-to-noise ratio (SNR) and optimize the extracted ROI through multichannel fusion for accurate HR measurement. The experimental results highlight that the method achieved lower errors under controlled lighting and moderate motion on PURE and custom datasets while maintaining consistent accuracy in less-constrained environments. Its overall performance remained stable across participants with different skin-tones, although minor errors are observed in individuals with dark skin-tones due to the melanin-induced attenuation of reflected light. These findings reflect the advantages of signal quality-aware weighted filtering and validate the use of residual-based correction mechanisms for improving the rPPG signal quality. There are still few limitations, such as limited datasets, extreme motion conditions while subjects are doing different activities. In future, this study will collect more dataset including subjects from different demographic areas consisting wide-range of skin tone variation, age differences and gender under varied lighting and dynamic motion scenarios. Furthermore, to improve proposed signal processing framework this study will implement deep neural network algorithms using self-supervised learning. Further studies will explore motion compensation algorithms and optical flow analysis to analyze depth information for monitoring blood volume pulse changes under dynamic motion conditions. Additionally, skin-tone adaptation approaches and frame-wise reflectance estimation will be investigated to reduce performance errors in melanin-induced skin variations. Also, clinical trials will be conducted through multi-sensor fusion strategies such as RGB and NIR fusion for real-time implementation in healthcare facilities and nonclinical applications.
